# The effects and mechanism of taxanes on chemotherapy-associated ovarian damage: A review of current evidence

**DOI:** 10.3389/fendo.2022.1025018

**Published:** 2022-11-24

**Authors:** Chuqing Wu, Tong Wu, Dan Chen, Simin Wei, Weicheng Tang, Liru Xue, Jiaqiang Xiong, Yibao Huang, Yican Guo, Ying Chen, Meng Wu, Shixuan Wang

**Affiliations:** ^1^ Department of Obstetrics and Gynecology, Tongji Hospital, Tongji Medical College, Huazhong University of Science and Technology, Wuhan, Hubei, China; ^2^ National Clinical Research Center for Obstetrical and Gynecological Diseases, Wuhan, Hubei, China; ^3^ Key Laboratory of Cancer Invasion and Metastasis, Ministry of Education, Wuhan, Hubei, China; ^4^ Department of Obstetrics and Gynecology, Zhongnan Hospital of Wuhan University, Wuhan, Hubei, China

**Keywords:** taxanes, chemotherapy, ovarian function, amenorrhea, fertility preservation

## Abstract

Chemotherapy is often a cause of premature ovarian insufficiency and infertility since the ovarian follicles are extremely sensitive to the effects of chemotherapeutic agents. Different chemotherapeutic agents with varying mechanisms of action may damage ovarian function differently. Taxanes are widely used in clinical cancer treatment, but the specific reproductive toxicological information is still controversial. This review described the impact and duration of taxanes on ovarian function in women and analyzed the possible reasons for different conclusions. Furthermore, the toxicity of taxanes on ovarian function and its possible mechanisms were discussed. The potential protective strategies and agents against ovarian damage induced by taxanes are also reviewed.

## Introduction

The global burden of cancer has been increasing in recent years, showing a trend towards onset at a younger age. There were approximately 19.3 million new cancer cases worldwide in 2020, and the number is expected to reach 28.4 million by 2040 (1). Fortunately, with the development of early diagnosis and multimodality treatments, the survival rates of cancer patients have improved significantly (2). Therefore, increasing attention has been paid to the quality of survival and the risk of long-term sequelae. Chemotherapy-associated ovarian damage (CAOD) is a well-recognized sequela in females with cancer. It has been associated with a higher incidence of premature ovarian insufficiency (POI), leading to delayed puberty, infertility, and disease associated with secondary chronic estrogen deficiency, such as osteoporosis, cardiovascular dysfunction, and Alzheimer’s Disease (3, 4). These consequences contribute to poor health and affect psychological and social well (5). By 2025, approximately 100 million women will be at risk of CAOD worldwide (6). Hence, understanding the biological mechanisms of ovarian damage caused by chemotherapeutic agents and developing new preservation strategies becomes paramount.

Chemotherapeutic agents include alkylating agents, alkylating-like platinum complexes, anthracyclanes, taxanes, topoisomerase inhibitors and vinca alkaloids. As a broad-spectrum anticancer drug, taxanes are used as systemic chemotherapy drugs, such as in breast, ovarian, lung, bladder, and other types of solid tumors cancer treatment. Nowadays, the taxanes family can be divided into three groups: paclitaxel, the synthetic derivatives of paclitaxel (docetaxel and cabazitaxel), nanoparticle albumin-bound paclitaxel (7, 8). Taxanes alter multiple cellular oncogenic processes, including mitosis, angiogenesis, apoptosis, inflammatory response, and oxygen species (ROS) generation, which result in cell death (9).

The detrimental consequences of taxanes chemotherapy on ovarian function have long been documented in clinical and animal experiments. Assessing the specific gonadotoxicity induced by taxanes is challenging, as they are frequently used sequentially or concurrently with other gonadotoxic drugs in clinical. Early clinical studies showed that adding taxanes to anthracycline-based chemotherapy had no additional adverse effect on the menstrual cycle in women younger than 40 years old. In contrast, a large scales prospective cohort found that adding taxanes to anthracycline-based chemotherapy increased the probability of amenorrhea (10). Then, late-phase studies using precise indicators of ovarian reserve function reported evidence of ovarian toxicity induced by taxane-based combined chemotherapy (11, 12). In addition, two recent clinical studies showed that taxanes monotherapy had a strong ovarian toxic effect by assessing amenorrhea and serum steroid hormone levels in premenopausal patients (13–15).

Animal studies exploring paclitaxel’s effects on CAOD also showed divergent results. Both in rats and mice models, a high dose of paclitaxel (7.5 mg/kg) treatment caused the depletion of primordial follicular reserve (16, 17). In contrast, some studies in rodent models reported no effects on the number of follicles at pre-antral stage after repeated administration of paclitaxel; only the antral follicles decreased significantly, indicating that paclitaxel’s ovarian toxicity is mild and transient (18, 19). In addition, *in vitro* culture of neonatal mouse ovaries shows that docetaxel adversely affects early growing follicles (20). *In vitro* culture of early secondary follicles, high concentration paclitaxel had detrimental impacts on the dynamics of follicle development (21). Furthermore, oocytes exposed to paclitaxel showed defective spindle organization and aneuploidy formation, especially in MII stage oocytes (19). Overall, the effect of taxanes on ovarian function remains controversial; more in-depth studies are needed to clarify its ovarian toxicity in humans and animals.

The mechanisms behind CAOD induced by taxanes chemotherapy have been extensively investigated, but they remain largely unclear. There is some evidence in animal models proved that taxanes may damage ovarian function through abnormal cell division (22), oxidative stress (OS) (23), and caspase-dependent apoptosis (20). Based on the mechanisms of ovarian damage, some possible compounds have been proposed to prevent taxanes’ gonadotoxicity (17, 19, 22, 23). This review aims to elucidate the impact of taxanes on ovarian function in clinical and animal studies. In addition, we also discussed the underlying mechanisms of taxanes-induced ovarian damage and the promising strategies for fertility preservation.

## The effects of taxanes on female ovarian function

The results of taxanes on ovarian function are mostly reported in women with breast cancer. Taxanes, combined with other chemotherapeutics, is widely used for breast cancer and significantly improves the disease-free and overall survival rates (24). However, the ovarian function of patients receiving taxanes chemotherapy remains uncertain.

### The effects of taxanes on levels of ovarian steroid hormone

Steroid hormonal and ultrasound tests, including anti-mullerian hormone (AMH), estradiol (E2), follicle-stimulating hormone (FSH), inhibin B, and antral follicle count (AFC), are commonly used to evaluate ovarian function (25). We summarized the published clinical studies on the effects of taxanes alone or combined with other chemotherapy agents on levels of ovarian steroid hormone.

The ovarian toxicity of taxanes monotherapy was best illustrated by its effects on ovarian function. Furlanetto et al. found that 57% of premenopausal patients with early breast cancer suffered from chemotherapy-induced ovarian failure (CIOF) after taxanes monotherapy, defined as FSH>12.4 IU/L, E2<52.2 ng/L, amenorrhea, and AMH level <0.22 ng/mL after treatment. The incidence of CIOF with taxanes alone was 55.6% at six months, 33.3% at 12 months, and 25% at two years. Furthermore, the median number of AFC was extremely low at the end of treatment and did not appreciably improve during two years of recovery (13). Together, the above results showed that taxanes monotherapy decreased ovarian reserve, but more studies are needed to confirm this conclusion.

Taxanes were often used in combination with other chemotherapy drugs, especially the anthracycline-based regiment, which includes AC (doxorubicin, cyclophosphamide), EC (epirubicin, cyclophosphamide), FAC (fluorouracil, doxorubicin, cyclophosphamide) and FEC (fluorouracil, epirubicin, cyclophosphamide) (26). Seven case-control trials about the changes of hormonal levels when patients received anthracycline based regimens with or without taxanes are presented in **Table 1** (11, 12, 27–30). Notably, Reh et al. showed that when women with breast cancer of stages I-IIIA received AC or AC followed by paclitaxel treatment, there were no significant differences in serum FSH or E2 levels at a mean of 28 months after chemotherapy (27). Perdrix et al. reported that the median AMH levels were lower in patients who received FEC sequentially with docetaxel than in patients who received FEC chemotherapy alone in women below 35 years with early breast cancer, indicating the combined use of paclitaxel increased the risk of ovarian damage. Besides, the AMH level did not restore to the age-matched level even after three to five years (30). In all, there is no consensus on whether adding taxanes to the AC regimen leads to a worse damage to ovarian endocrine function.

**Table 1:** The changes of female hormone levels with taxane-based chemotherapy.

Study	Breast cancer diganosis	Treatment regimen and Number of patients	Age (y)	Median follow up time	The level of serum steroid hormone with and without taxane use		
					AMH (*P* value)	E2 (*P* value)	FSH (*P* value)
Al-Rawi et al. (11)	I-III	4c AC→4c T (n=28) 4c AC (n=30)	39 (25-45)	End of treament (EOT)	AC→T (0.06 ng/ml); AC (0.06 ng/ml) P> 0.05	AC→T (6.88 pg/ml); AC (14.49 pg/ml) P< 0.016	N/A
Reh et al. (27)	I-IIIA	AC→T (n=6) AC (n=5)	40 (37-44)	28 (15-86)	N/A	AC→T (95.3 mIU/ml); AC (45.2 mIU/ml) P> 0.05	N/A
Yoo et al. (28)	I-III	4c AC→4c T (n=77) 4c AC (n=103)	43 (30-52)	6	N/A	E2↓ P=0.02	FSH↑ P=0.004
Arslan et al. (29)	N/A	AC→T (n=67) AC (n=19)	AC→T (34.8) AC (34.5)	AC→T 32 AC 25.4	N/A	AC→T (73.5 pg/ml); AC (39.5 pg/ml) P >0.05	AC→T (21.2 mIU/ml); AC (11.8 mIU/ml) P >0.05
Perdrix et al. (30)	N/A	3c FEC→3c D (n=45)	31.5 (11-35)	12	FEC→D (0.09 ng/ml); FEC (0.39 ng/ml) P=0.007	N/A	N/A
		6c FEC (n=9)					
Lambertini et al. (12)	I-III	3c FEC→3c D (n=127)	35.5 (31.5-38)	12	FEC→D (0.04 µg/L); FEC (0.22 µg/L) P=0.0006	N/A	N/A
		6c FEC (n=21)		36	FEC→D (0.18 µg/L) FEC (0.06 µg/L) P> 0.05	N/A	N/A

AC, anthracycline/cyclophosphamide; FEC, fluorouracil/epirubicin/cyclophosphamide; D/DTX, docetaxel; T/PTX, paclitaxel; c, cycle; N/A, not available; DPC, (Diagnostic Products Corporation, Los Angeles, CA).

Multiple reasons may cause the uncertainty of ovarian damage induced by taxanes. First, some clinical data do not provide clear information about the ovarian function of patients while receiving taxanes chemotherapy, which may have essential impacts on evaluation results. In addition, the sensitivity of the AMH detection assay needs to be considered. AMH levels in most studies were undetectable or close to the detection limits due to their limited sensitivity, which may result in no difference between different chemotherapy groups (11). A considerable AMH decline related to adding taxanes to the AC regimen by employing an automated AMH immunoassay method (30). Indeed, since there is no international standard for AMH, all related data should be interpreted cautiously. Second, the median age of patients was around 40 years, when the ovarian reserve was gradually decreasing and sensitive to chemotherapy damage (31). Lastly, heterogeneity varies between studies, including small population size, high missed follow-up rates, and scarce studies with taxanes alone. Therefore, extensive prospective studies are needed in the future to ascertain the impact of taxanes on ovarian reserve.

### The effects of taxanes on female menstrual cycle

The incidence of chemotherapy-induced amenorrhea (CIA) was easy to record and commonly used as an endpoint to evaluate taxanes’ reproductive toxicity. Iwamoto et al. reported that 69.4% of patients (138/199) with breast cancer of stages I-III had no menstrual cycle for at least six months after taxanes monotherapy (eight cycles of docetaxel or paclitaxel alone) (14). However, taxanes monotherapy is not a standard treatment, and most studies explored its ovarian function when taxanes were used in combination with chemotherapeutic agents. Ruddy et al. showed that breast cancer patients treated with adjuvant paclitaxel and trastuzumab had a relatively low amenorrhea rate (28%) in HER2-positive breast cancer patients at a median age of 44 years (32). The available data indicate that trastuzumab is unlikely to be gonadotoxic (33, 34); the ovarian toxicity induced by the combined regimen may come from paclitaxel. The incidence of CIA varied across different taxanes combination regimens such as anthracycline, epirubicin, and cyclophosphamide is shown in **Table 2** (14, 15, 27–29, 33, 35–43). Petrek et al. evaluated the ovarian function in women with a history of breast cancer of stages I-IIIA who received the standard therapy of AC alone or in combination with paclitaxel or docetaxel. The CIA rate drastically increased one month after the treatment, ranging from 10% to 30%. Patients with an AC regimen had the lowest CIA rate, and adding docetaxel to the AC regimen had the highest CIA rate (10). Sukumvanich et al. recruited 245 women with stage I to III breast cancer and prospectively examined the CIA rates after chemotherapy. They observed that AC sequential paclitaxel regimens caused a higher CIA rate than AC regimens at six months of follow-up (45% vs 37%) (38).

**Table 2:** The incidence of CIA with taxane-based regiment.

Study	Breast cancer diganosis	Treatment regimen (Number of patients)	Age(y)	CIA definition(m)	Follow-up (m)	The incidence of CIA with andwithout taxane use (%)	*P* value
Reh et al. (27)	I-IIIA	AC→T (n=28) AC (n=17)	N/A	≥6	6	AC→T (96); AC (82)	P > 0.05
					28	AC→T (35.7); AC (9.1)	P < 0.05
Yoo et al. (28)	I-III	4c AC→4c T (n=120) 4c AC (n=192)	43(30-52)	Long-term CIA ≥12 and not recovery	17.5	AC→T (64.2); AC (53.6)	P < 0.05
				Temporary CIA ≥3 and recovery	17.5	AC→T (34.2); AC (37.5)	P > 0.05
Najafi et al. (15)	I-IV	4c AC→4c T (n=75) 4c AC/6cFAC (n=111)	40(25-56)	≥3	36	AC→T (78.7); AC or CAF (66.7)	P < 0.05
Iwamoto et al. (14)	I-III	4c AC→4c T (n=90) 4c AC→4c D (n=105) 8c PTX (n=94) 8c DTX (n=105)	44.2(24-62)	≥6	60	AC→T (76.9); AC→D (75.2); PTX (62.8); DTX (75.2)	P > 0.05
Okanami et al. (35)	I-III	AC/FAC→T (n=49) AC/FAC (n=17)	37(26-40)	Without menstruation during chemotherapy	27.6	AC→T (93.9); AC (70.6)	P < 0.05
				Persistent CIA≥12	27.6	AC→T (24.5); AC (11.8)	P > 0.05
Abusief et al. (33)	Early stage	AC→T (n=203) AC (n=228)	43(25-55.6)	≥6	33	AC→T (56.6); AC (54.8)	P >0.05
Turnbull et al. (36)	Early stage	3c FEC→3cD (n=66) 3c FEC (n=41)	43 (35-50)	Without menstruation during follow-up	60	FEC→D (77); FEC (76)	P > 0.05
Davis et al. (37)	N/A	AC→T (n=43) FAC→T (n=4) CMF→T (n=6) AC (n=59) FAC (n=14) CMF (n=33)	40.8(18-50)	≥12	N/A	AC→T/FAC→T/CMF→T (43.4); AC/FAC/CMF (51.9)	P > 0.05
Sukumvanich et al. (38)	I-III	AC→T (n=143)	38.5(20-45)	≥6	6	AC→T (45.4); AC (37.4)	P > 0.05
		AC (n=111)			12	AC→T (29.4); AC (19.4)	P > 0.05
					24	AC→T (23.7); AC (15.1)	P > 0.05
Berliere et al. (39)	I-III	3c FEC→3c D (n=70) 6c FEC (n=84)	43.5(28-58)	Without menstruation during chemotherapy	End of treatment	FEC→D (93); FEC (92.5)	P > 0.05
Tham et al. (40)	N/A	4c AC→3m T (n=117) 4c AC (n=74)	≤50	≥6	N/A	AC→T (61); AC (44)	P < 0.05
Abdel-Razaq et al. (41)	I-III	4c AC→4c/12c T (n=13) 4c AC→4c D (n=10) 4c AC (18)	35.7(22-44)	≥12	≥36	AC→T (69.2); AC→D (66.7); AC (38.9)	P < 0.05
Arslan et al. (29)	N/A	AC→T (n=67) AC (n=19)	AC→T (34.8) AC (34.5)	Without menstruation during follow-up	AC→T 32 AC 25.4	AC→T (67.2); AC (42.1)	P < 0.05
Narmadha et al. (42)	I-III	6c FEC (n=8) 6c DEC (n=6) 6c FAC (n=28) 6c DAC (n=8)	40 (26-50)	≥3	N/A	DAC/DEC (100); FAC/FAC (75)	P < 0.05
Zhou L et al. (43)	I-III	4c FEC→4c/6cT(n=18) 6c FEC/FAC (n=85)	45.4 (26-57)	Without menstruation during follow-up	4	FEC→4c/6c T (61.1); FEC/FAC (70.6)	P > 0.05

AC, anthracycline/cyclophosphamide; FEC, fluorouracil/epirubicin/cyclophosphamide; FAC, fluorouracil/doxorubicin/cyclophosphamide; CMF, cyclophosphamide/methotrexate/fluorouracil; EC,epirubicin/cyclophosphamide; D/DTX, docetaxel; T/PTX, paclitaxel; c,cycle; N/A not available.

There is conflicting evidence regarding taxanes aggravated the gonadotoxicity of other cytotoxic drugs. In Turnbull’s study, patients with early-stage breast cancer showed no difference in CIA rates at a median follow-up of 60 months after anthracyclines-based or anthracyclines-taxane-based chemotherapy (36). A meta-analysis published in 2014 regarded that taxane-based regimens significantly increased the rate of CIA regardless of the definition of CIA (44). Another meta-analysis described that when anthracycline-based chemotherapy combined with taxanes was not related to a higher risk of CIA, but the level of evidence was weak (45). In the latest meta-analysis, Wang et al. reported that the addition of taxanes to anthracycline-based regimens would significantly increase the CIA rates with no heterogeneity and publication bias (46).

Based on the above studies, the effects of taxanes on the female menstrual cycle are unclear. Possible explanations for this discrepancy may be due to the following points. First, the definition of CIA and the follow-up duration varied across studies (**Table 2**). The incidence of CIA decreases as ovarian function gradually recovers, which may cause different results regarding taxane-induced ovarian damage. Han et al. followed patients’ menstrual cycles for up to three years, and a higher CIA rate was observed in taxane-based regimens compared with non-taxane-based regimens within the first year. No significant differences were observed after the second year (47). Second, the reproductive toxicity of taxanes is affected by their types, duration, and dosage. For example, docetaxel appears to have higher ovarian toxicity than paclitaxel (10). Third, a higher CIA rate is associated with the increasing female age. Tham et al. found that women younger than 40 who received AC sequentially paclitaxel therapy had a higher CIA rate compared with those receiving AC alone. However, these differences were not statistically significant in women older than 40 (40). Fourth, many patients were treated with tamoxifen for a long time, and the use of tamoxifen appears to be associated with higher rates of CIA (46). Consequently, the reported CIA rates may be overestimated. Last, CIA is also affected by the menstrual cycle phase when chemotherapy begins. Women had a higher incidence of CIA when patients received chemotherapy during the follicular development phase than in other phases (48).

The long-term impact of taxanes on gonadotoxicity should also be given attention. Some clinical evidence showed that taxanes-induced amenorrhea had a better recovery rate during long-term follow-up, suggesting that ovarian damage induced by taxanes may be temporary. In a recent study, approximately 66.7% of patients with breast cancer receiving taxanes monotherapy recovered from CIA, and more than 70% restored E2 and FSH after two years after treatment (13), which suggested that ovarian damage induced by taxanes may be temporary. This is probably because human follicles are periodic cycled; it takes approximately six months for dormant follicles to develop into ovulatory follicles (49). Another reason may be related to the rapid elimination of paclitaxel, with a mean elimination half-time only of 2.44 h (50). We summarized the percentage of menstruation restoration and the duration of amenorrhea in patients with or without the addition of taxanes chemotherapy in **Table 3** (15, 27–29, 35, 36, 39, 40, 42, 43). From the table, we found that contradicting results exist for the recovery and duration of CIA induced by taxane-based chemotherapy. The recovery of ovarian function after chemotherapy means that women have a chance to become pregnant, but it is with regret that fertility is rarely evaluated in clinical trials. Hamy et al. reported that women treated with anthracyclines and cyclophophamide-based regimens were more likely to get pregnant than taxane-based regimens (51). In short, CIA induced by taxanes may be transient and long-term effects are relatively small, while further studies are needed to confirm this conclusion.

**Table 3:** The percentage of menstruation restoration after taxane-based chemotherapy.

Study	Breast cancer diganosis	Treatment regimen and Number of patients	Age (y)	Follow-up(m)	Menstrution restoration (%)	*P* value
Yoo et al. (28)	I-III	4c AC→4c T (120) 4c AC (192)	43 (30-52)	17.5 (7.5-29.2)	AC→T (34.7); AC (41.1)	P > 0.05
Najafi et al. (15)	I-IV	4c AC→4c T (75) 4c AC or 6c FAC (111)	40 (25-56)	36 (12-120)	AC→T (49.1); AC or FAC (27.1)	P < 0.05
Reh et al. (27)	I-IIIA	AC→T (28) AC (17)	N/A	28.2 (15-86)	AC→T (64); AC (57)	P > 0.05
Okanami et al. (35)	I-III	4c AC→4c T (49) 4c AC (17)	37 (26-40)	27.6 (10.6-64.8)	AC→T (75.5); AC (88.2)	P > 0.05
Turnbull et al. (36)	Early stage	3c FEC→3c D (66) 6c FEC (41)	43 (35-50)	60	FEC→D (44); FEC (32)	P >0.05
Berliere et al. (39)	I-III	3c FEC→3c D (70) 6c FEC (84)	43.5 (28-58)	12	FEC→D (35.5); FEC (23.7)	P < 0.05
Narmadha et al. (42)	I-III	6c FEC (n=8) 6c DEC (n=6) 6c FAC (n=28) 6c DAC (n=8)	40 (26-50)	N/A	DEC/DAC (35.8); FEC/FAC (51.9)	P < 0.05
Tham et al. (40)	NA	4c AC→3m T (n=117) 4c AC (n=74)	N/A	N/A	AC→T (37.8); AC (29.2)	P > 0.05
Arslan et al. (29)	NA	AC→T (n=67) AC (n=19)	AC→T (34.8) AC (34.5)	AC→T 32 AC 25.4	AC→T (82.2); AC (100)	P < 0.05
Zhou L et al. (43)	I-III	6c FEC/FAC (n=85) 4c FEC→4c/6c TEC (n=18)	45.44 (26-57)	N/A	AC→T(27.8); AC (20.0)	P > 0.05

AC, anthracycline/cyclophosphamide; FEC, fluorouracil/epirubicin/cyclophosphamide;FAC, fluorouracil/doxorubicin/cyclophosphamide; CMF, cyclophosphamide/methotrexate/fluorouracil; EC,epirubicin/cyclophosphamide; D/DTX, docetaxel; T/PTX, paclitaxel; c,cycle; N/A not available; m, month.

## Animal studies about the effects of taxanes on ovarian function

Understanding taxanes’ ovarian toxicity is essential for developing ovarian preservation approaches. Here, we summarized animal studies associated with the effects of taxanes on ovarian fertility and endocrine function.

### The effects of taxanes on follicular quantity

Ovaries contain follicles of various stages, including primordial, primary, secondary, antral, and preovulatory follicles. The following data obtained in animal models demonstrated that taxanes significantly affect the number of follicles. A single intraperitoneal injection of high dosage paclitaxel at 7.5 mg/kg significantly decreased the number of primordial follicles after one week of exposure (16, 17, 23, 52). However, in some studies, paclitaxel decreased antral follicles and increased atretic follicles, but did not affect the number of primordial follicles (18, 19). The main concern regarding ovarian damage is whether it significantly affects the primordial follicle pool because it is non-renewable, and may lead to POI. The loss of primordial follicles may attribute to direct injury to the primordial pool or an indirect outcome of the accelerated primordial follicle activation due to a major loss of mature follicles, known as the burnout model (53). In contrast, Nicosia et al. reported that mature follicles were more prone to damage induced by chemotherapy than immature follicles (54). A recent study also showed single or repeated intraperitoneal injection of high dosage paclitaxel at 30 mg/kg decreased the number of antral follicles without reducing primordial follicles; and the reduction only maintained for 1-2 estrous cycles, suggesting that the reproductive toxicity of paclitaxel was mild and transient (19). Similarly, *in vitro* intervention of neonatal mouse ovaries, docetaxel reduced early growing follicles without affecting primordial follicles (20). The possible reason is that taxanes act on actively dividing proliferating cells, and the growing follicle is in a stage of rapid development. Furthermore, the reduction in follicle number induced by taxanes was concentration-dependent both *in vitro* and *in vivo*, and was usually observed at high concentrations but not at low concentrations (16, 20). Based on the above results in animals, Taxanes can reduce the number of follicles *in vitro* and *in vivo*, but there is no consensus on which type.

### The effects of taxanes on follicles quality

The damage of taxanes to ovaries can be further studied in animals by detecting involved indicators of follicle quality. Severe follicular damage was observed even at low-dose docetaxel (0.1µM) treatment *in vitro* (20). Granulosa cells of growing follicles are the first cellular target of docetaxel-induced follicular damage, and oocyte damage followed as a downstream consequence of granulosa cells compromised (55). Approximately 30% of abnormal transitional follicles and more than 80% of abnormal primary follicles were manifested as eosinophilic, shrunken, heterogeneous cytoplasm or condensed nuclear chromatin. In another study, mouse pre-antral follicles were treated with 10^–10^ M paclitaxel for five days *in vitro*, the follicular survival and growth were significantly suppressed, and no ovulation was observed. The follicle survival rates decreased by approximately 50% compared with controls, showing morphological abnormalities such as follicular constriction and oocyte extrusion. Furthermore, the expression of follicle development-relevant genes, growth differentiation factor 9 (GDF9), and bone morphogenetic protein 15 (BMP15) were also repressed by paclitaxel (56). Another study investigated the effects of paclitaxel on early secondary follicles of mouse and treated these follicles with 2.5 × 10^−10^, 2.5 × 10^−9^, and 2.5 × 10^−8^ M paclitaxel for 12 days (21). The results showed that high concentrations of paclitaxel inhibited the growth of secondary follicles, which is consistent with the above study by Kim et al. (56). Furthermore, a recent animal study demonstrated that a high concentration of paclitaxel affects the quality of MI and MII stage oocytes *in vitro*, with disordered spindle organization, decreased maturation, increased aneuploid oocytes, and lower fertilization rate (19).. Based on the above results, taxanes treatment damages ovarian granulosa cells and oocytes, leading to follicular death or aneuploidy.

### The effects of taxanes on the ovarian stroma

Ovarian stroma, typically the supporting tissue of follicles, includes stromal cells, immune cells, blood vessels, lymphatic vessels, nerves, and extracellular matrix components (57). The ovarian stroma has adverse effects on the health of the ovarian reserve, affecting normal follicle development (58). Early in 2007, the ovarian cortex of chemotherapy patients exhibited blood vessel damage and fibrosis (59). Cyclophosphamide, busulphan and doxorubicin have been reported to cause vascular and stromal damage in the ovary (58, 60, 61), which might impair ovarian function. However, the effects of taxanes on ovarian stroma have been less studied by previous scholars. A study on the time accumulation of the drugs showed that doxorubicin accumulated first in the ovarian stroma’s core because of its close relationship to the blood supply (62). Some clinical evidence revealed that young breast cancer patients receiving taxane-based chemotherapy had ovarian vascular damage, with decreased ovarian blood flow and reduction in ovarian size at the end of treatment (63). Then, the same group reported the continuous prospective evaluation of ovarian function in these patients. They indicated that ovarian toxicity might derive from acute ovarian vascular damage and the ovarian blood flow was partially restored at long-term follow-up (64). In another animal study, docetaxel negatively affected ovarian stromal cells with the high expression of apoptotic indicators, including cleaved caspase 3, cleaved caspase 8, Bax and cleaved poly (ADP-ribose) polymerase (20). Future studies are warranted to further assess the role of taxane-induced vascular toxicity in the ovary.

### The effects of taxanes on ovarian endocrine and fertility

Endocrine and fertility, as two main functions of the ovary, need to be taken seriously when considering the impact of taxanes. Chen et al. reported that paclitaxel produced an inhibitory effect on basal progesterone (P4) and E2 in a dose- and time- dependent manner in porcine ovarian granulosa cells (65). Nevertheless, Tarumi et al. showed that the serum E2 level was slightly lower than that of the control group after repeated paclitaxel injections in rats. At the same time, there was no difference in the P4 level (18). Furthermore, the mating experiment was performed to evaluate the consequence of taxanes on female fertility. Tarumi et al. showed that rats significantly decreased fetuses and implantations rate when mated immediately after administration. Still, these adverse effects were not detected when mated 24 days after administration (18). In another research, paclitaxel contributed to a decrease in pregnancy rates and an increase in stillbirths. Besides, the karyotypes of the offspring were normal, indicating that the damage of paclitaxel to the offspring is embryonic lethal (19). In addition, *in vitro*–fertilized (IVF) embryos culture found that paclitaxel treatment resulted in lower cleavage and blastocyst development rates of bovine embryos (66). Overall, paclitaxel disturbed the secretion of endogenous ovarian hormone and fertility, but the damage may be transient and reversible.

## The mechanism of taxane-induced ovarian damage

**Figure 1:**
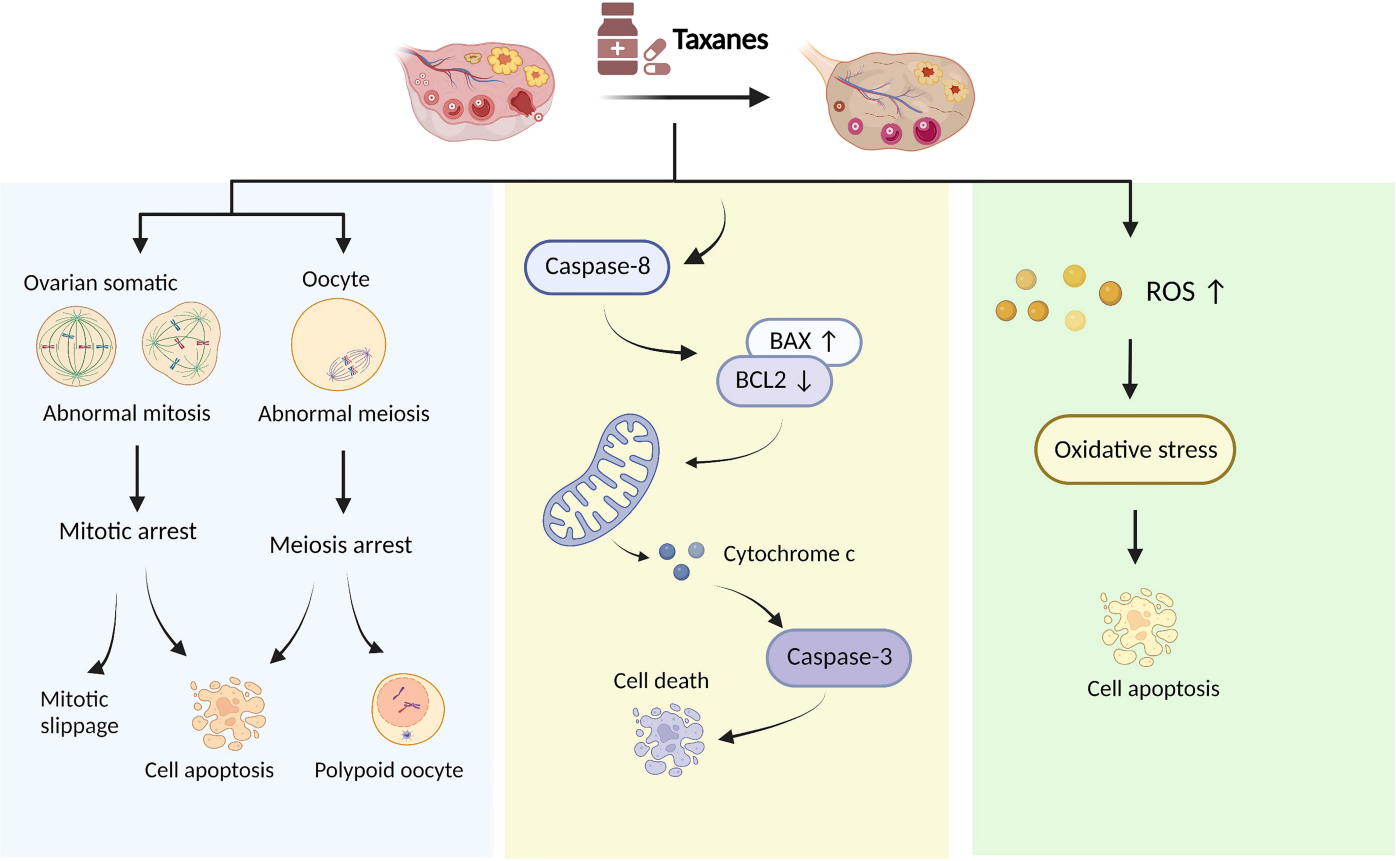
Proposed taxanes mechanisms of action in ovarian damage.

The primary pharmacological mechanism of taxanes was to promote microtubule assembly and resist depolymerization (67). Early studies indicated that cancer cells treated with taxanes cannot establish a normal mitotic apparatus and were arrested in the late G2/M phase of the cell cycle (67, 68). Further studies revealed that taxanes influence multiple processes in cancer cells, including mitosis, apoptosis (69), oxidative stress (70), and inflammatory response (71). However, the mechanism of ovarian damage caused by taxanes is unclear; current studies have shown that abnormal cell division, follicular cell apoptosis, and oxidative stress might be involved in the damage to ovarian function induced by taxanes, as shown in **Figure 1**.

### Taxanes cause abnormal cell division of follicular cells

The follicle is composed of an oocyte surrounded by granulosa and thecal cells. Oocytes complete their first meiotic division before ovulation with the extrusion of the first polar body, during which they are more vulnerable to chemotherapy. Oocytes can be divided into the germinal vesicle (GV), germinal vesicle breakdown (GVBD), metaphase I (MI), and metaphase II (MII) phase according to the difference in the nucleus (71). In a mice model that received paclitaxel intraperitoneally immediately after injection of human chorionic gonadotropin (HCG) to promote ovulation, Mailhes JB et al. found that paclitaxel blocked the meiosis of oocytes with increased MI phase oocytes, diploid oocytes and polyploid zygotes (72). Similarly, oocyte with abnormal meiotic status was observed with high concentrations of paclitaxel (30mg/kg) treatment in mice (19). In addition, GV stage oocytes are impervious to the harmful effects of paclitaxel because microtubules have not yet assembled into a specific format at this stage (73), and MI and MII oocytes are vulnerable to paclitaxel since their maturation relies on the assembly of microtubules and the spindle formation (74, 75). These results suggested that paclitaxel affects the meiotic process of oocytes, especially in MI and MII oocytes. Ovarian granulosa cells are a kind of mitotically active cells located at the outermost layer of the follicle; thus, they are sensitive to chemotherapy. Cell cycle analysis has been performed on granulosa cells after 6, 12, 24, and 48 hours of paclitaxel treatment. These results showed that paclitaxel produced the characteristic G2 block at 12h (20%) and 24h (35%), which increased at 48h up to nearly 100%. In addition, the G2 cyclins A and B1, and their partner CDK1 were down-regulated at 48h paclitaxel exposure (22). Collectively, paclitaxel as a microtubule-targeting drug affects mitosis in granulosa cells and meiosis in oocytes.

### Taxanes cause the apoptosis of follicular cells

Follicular apoptosis is a pivotal event in the depletion of follicles in chemotherapy-treated women (76, 77). Many studies have demonstrated that taxanes induce the apoptosis of follicular cells. Lopes et al. found that apoptosis-associated markers cleaved caspase 3 and cleaved caspase 8 expressed extensively in ovarian granulosa cells and stromal cells after low-dose docetaxel intervention *in vitro* (20). Moreover, the expression of anti-apoptosis genes Bcl2 and XIAP was significantly down-regulated in granulosa cells by the treatment with 10 ^-10^ M paclitaxel *in vitro* (56). XIAP is a known inhibitor of apoptosis protein 3 (IAP3), and overexpression of XIAP significantly improves the survival of pre-antral follicles (78). PI staining and TUNEL assay indicated a large proportion of cells underwent apoptosis after 48h of culture with paclitaxel in rat primary granulosa cells (22). Similarly, paclitaxel-induced follicular apoptosis was also observed in mice with high cleaved caspase 3 expression in granulosa cells (23). Lopes et al. indicated that docetaxel activated the mitochondria-dependent apoptotic pathway in ovarian granulosa cells, resulting in the upregulation of Bax and cytochrome C movement from mitochondria to the cytoplasm. The cytochrome C in the cytoplasm subsequently stimulated downstream effector caspases such as caspase 3, leading inactivation of cellular DNA repair followed by apoptosis (20). Indeed, paclitaxel-induced cell apoptosis was inextricably linked to cell cycle arrest. The checkpoint of mitotic spindle assembly and aberrant activation of cyclin-dependent kinases were shown to be involved in paclitaxel-induced apoptosis (79). Besides, G2 arrest of granulosa cells occurred after paclitaxel intervention for 12h, whereas apoptosis was evident only after 48h, indicating that apoptosis may be secondary to G2 arrest (22). Together, advances in apoptosis research have extended our understanding of the mechanisms of paclitaxel-induced follicular cell damage. However, the downstream biochemical events from paclitaxel’s binding to microtubules that lead to follicular cell apoptosis are poorly understood.

### Taxanes cause oxidative stress in ovaries

OS is a condition wherein pro-molecules, including ROS and nitrogen species (NOS), and antioxidant defence are out of balance. OS significantly negatively impacts ovarian cells and oocyte health (80). Excessive ROS accumulation leads to OS when ovaries are exposed to chemotherapeutic agents, γ-radiation, polycyclic aromatic hydrocarbons, or a poor lifestyle (80). For example, paclitaxel significantly induces mitochondrial ROS production by activating the STAT3 signalling pathway (81). ROS production is essential to paclitaxel cytotoxicity and is an early step before paclitaxel-induced cancer cell apoptosis (70). Qin et al. proposed that the loss of primordial follicles caused by paclitaxel may be related to OS. 4-hydroxynonenal (4-HNE), an established biomarker of OS, was significantly increased in oocytes and granulosa cells of paclitaxel-treated mice, especially in primordial follicles (23). These studies suggested that taxanes may generate oxidative metabolites, which increase OS and consequently triggers apoptosis in the ovary; however, it may be too early to draw a conclusion (82).

## Protective approaches to ovarian damage during taxanes therapy

**Figure 2:**
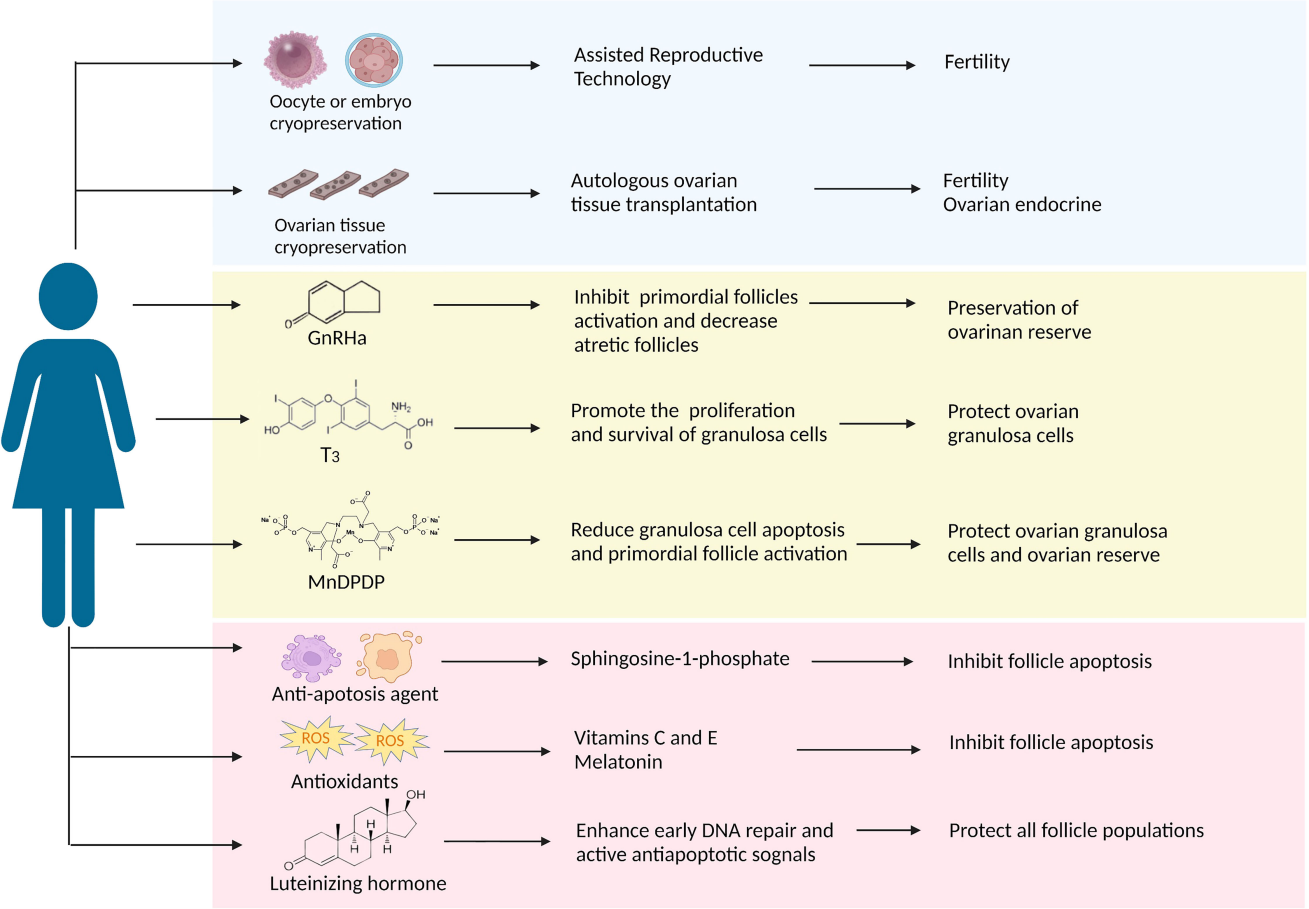
A summary of the protective strategies applied in clinical practice or have future potential against paclitaxel-induced ovarian damage. The approach discussed in this article is divided into three parts: the blue background box shows the methods that have been applied in clinical practice; the yellow background box shows potential strategies that have been proved to target taxane-induced ovarian damage in laboratory studies; the pink background box shows the potential strategies as presumed by the potential causative mechanism that can resist chemotherapeutic agents including taxanes.

Some women still hope to have children after chemotherapies with the delay of first births and the trend of younger patients (83). Recently, there has been extensive discussion about preserving fertility and quality of life in tumor survivors. The American Society of Clinical Oncology (ASCO) recommends that individuals should seek fertility preservation before cancer treatment and update practice guidelines regularly (84–86). Here, we aim to review several ovarian protective strategies against paclitaxel-induced damage in the following text and **Figure 2**.

### Oocyte, embryo, and ovarian tissue cryopreservation

Oocyte and embryo cryopreservation are currently considered standard practice according to the latest ASCO clinical practice guidelines, and ovarian cryopreservation is still an experimental method because of its immaturity (86). Compared to embryo cryopreservation, oocyte cryopreservation offers a better option for women without male partners. However, both techniques require ovarian stimulation to obtain oocytes, thereby delaying two weeks to six weeks in chemotherapy initiation. Fortunately, the problem is expected to be resolved. Von Wolff et al. developed an ovarian stimulation strategy in that patients received GnRH antagonists and recombinant FSH irrespective of the stage of the menstrual cycle, allowing oocyte collection within two weeks (87). Nowadays, random start ovarian stimulation is becoming more popular, and it does not appear to delay the onset of chemotherapy (88). Besides, it may increase the potential risk of hormone-sensitive tumors with short-term exposure to high estrogen levels, although there is no clear evidence (89). The advantage of ovarian tissue cryopreservation is restoring ovarian endocrine function after transplantation (90) and not requiring ovarian stimulation. In a meta-analysis, Pacheco et al. autologous included nineteen studies thought that autologous ovarian tissue transplantation (OTT) showed higher reproductive performance, the rates of live birth and ongoing pregnancy were 37.7%, and the recovery rate of endocrine was 63.9% (91). Many experts believe that adequate evidence currently exists to support the use of OTT as a feasible and valid technique and will become standard therapy within the next few years (92). Following ovarian tissue transplantation, the most significant concern would be the potential for the re-introduction of cancer cells, especially hematological malignancies. Therefore, ovarian tissue screening should be performed to detect cancer cells, and patients at high risk of ovarian involvement should be cautiously selected for ovarian tissue cryopreservation. Altogether, these advances in oocyte, embryo, and ovarian tissue cryopreservation have contributed to fertility preservation for cancer patients before chemotherapy. Regrettably, there are no relevant reports about the above methods of fertility protection before and after paclitaxel therapy.

### Gonadotrophin-releasing hormone analogs

GnRH analogs (GnRHa), comprising agonists and antagonists, produce a similar decline in GnRH secretion through different pathways (93). GnRHa inhibit the secretion of gonadotropins and prevent follicles development through the HPO axis, reducing chemotherapeutic drugs’ damage to actively growing follicular cells (94). The underlying mechanism of ovarian protection may be the reduction of ovarian blood perfusion induced by GnRHa, thereby reducing chemotherapeutic drug accumulation (95). In addition, GnRHa also reduce cell apoptosis by directly activating ovarian GnRH receptors or indirectly on peripheral cumulus cells (96). Another revolutionary speculation is that GnRHa protect undifferentiated germline stem cells and eventually generate *de novo* primordial follicles (75). Many clinical studies showing the positive effect of GnRHa on ovarian function in women with malignancies who received chemotherapy (97–99), but do not prove in statistically in the meta-analysis that GnRHa combined with chemotherapy reduces gonadal toxicity due to conflicting results and substantial heterogeneity (100–102). GnRHa are not recommended as a preferred alternative to proven fertility preservation methods, such as oocyte and embryo cryopreservation, according to ASCO (86) and ASRM guidelines (103).

An RCT study showed a significant reduction in early menopause rates when patients received intramuscular triptorelin at a dose of 3.75 mg at least one week prior to chemotherapy, including taxane-based regimens, and then repeated every four weeks during chemotherapy (104). It is worth noting that GnRHa needs to be injected 1-2 weeks before chemotherapy; delaying chemotherapy may lead to deterioration of the disease process and possibly to rapid recurrence. Many animal studies have demonstrated that GnRH agonists protect against paclitaxel-induced ovarian damage *in vitro* and *in vivo*. GnRH agonists (2.5 µg/d) were injected in advance for 28 days in rat models until ovarian suppression, and a single dose of paclitaxel (7.5 mg/kg) was administered. Follicle counts indicated that primordial follicles could be preserved after paclitaxel chemotherapy (17). In another experiment, GnRH agonists effectively suppressed follicle maturation and decreased atretic follicles during paclitaxel chemotherapy. Furthermore, GnRH agonists shorten the time of paclitaxel-induced MII oocyte damage persisted (19). In addition, GnRH agonists protect the ovary from docetaxel-induced damage with the reduction of the total follicle and double-strand DNA breaks (105). Overall, given the current evidence, GnRHa is the most promising drug for taxane-associated chemotherapy damage.

### Thyroid hormone

Thyroid hormones (THs), including L-triiodothyronine (T3) and L-tetraiodothyronine (T4), play an indispensable role in human growth and development (106). The cross-talk between the hypothalamic-pituitary-gonadal (HPO) axis and the hypothalamic-pituitary-thyroid (HPT) axis is vital in ovarian function (107). Abnormalities in the thyroid hormone can adversely affect female reproduction, causing menstrual disorders and infertility (108). T3 might have a direct role in ovarian physiology *via* its receptors that promote the proliferation and survival of ovarian granulosa cells (109, 110) and the development of pre-antral follicles (111, 112). About 40-60% of cells died after paclitaxel intervention by MTT and TUNEL assays, and T3 supplementation significantly reduced this ratio in rat primary granulosa cells. Besides, T3 could effectively overcome paclitaxel-induced G2 phase arrest, protecting granulosa cells from apoptosis and maintaining cell viability (22). Based on the above studies, thyroid hormone T3 is able to protect ovarian granulosa cells from paclitaxel-induced apoptosis, while more experiments need to be conducted for further verification.

### Mangafodipir

Mangafodipir (MnDPDP) is a chelate of a paramagnetic manganese (II) ion and of the ligand fodipir (DPDP, a vitamin B6 derivate), a superoxide dismutase (SOD) mimetic with peroxidase and glutathione reductase activities, which plays a role in multiple stages of the ROS cascade, protecting cells from H_2_O_2_-induced apoptosis (113, 114). So far, several studies have demonstrated that MnDPDP is helpful for some diseases caused by oxidative damage or oxidative damage resulting from certain drugs or physical therapies, such as adjuvant cancer chemotherapy, acute myocardial infarction, and liver ischemia-reperfusion injury (115). Qin et al. proposed that MnDPDP might ameliorate ovarian injury caused by paclitaxel-induced oxidative stress. They confirmed that MnDPDP could partially reduce paclitaxel-induced granulosa cell apoptosis and primordial follicle activation *via* its SOD activity without affecting the antitumor activity of paclitaxel (23). But due to the limited studies, the protective effects of MnDPDP on ovarian function during chemotherapy are unclear.

### Other candidates

Based on the mechanism of ovarian damage induced by taxanes, both antioxidants and anti-apoptotic agents are expected to play a protective role in ovarian function. Sphingosine-1-phosphate (S1P) is a metabolite of cell membrane sphingolipids that, as an anti-apoptotic agent, protects cells from ceramide-induced apoptosis (116). S1P has been shown to protect the ovary and preserve fertility from radiation and chemotherapy in mice and human ovary tissue *in vitro* (117, 118). Luteinizing hormone (LH), a steroid hormone that plays a cardinal role in follicular development and ovulation, has been proven to protect the ovarian reserve and ameliorate fertility during alkylating agents chemotherapy by generating anti-apoptotic signals and favoring DNA repair pathways in mice (119, 120). Further research found that some antioxidants, including vitamins C and E, melatonin, N-acetylcysteine, and coenzyme 10, also show ovarian protection during chemotherapy. Among them, melatonin, as a powerful antioxidant, prevents the loss of cisplatin-induced primordial follicles by inhibiting the overactivation of primordial follicles (121). Future explorations are needed to demonstrate their effectiveness in taxane-induced ovarian damage.

## Conclusion and future prospects

Based on the available evidence, the exact influence of taxanes on ovarian function in clinical is still uncertain because taxanes are frequently combined with other cytotoxic agents. Relevant animal studies reveal that taxanes affect the quantity and quality of follicles, leading to endocrine disruption and adverse fertility consequences. The taxanes-induced ovarian damage is closely associated with abnormal cell division, follicular cell apoptosis, and ROS accumulation. Targeted strategies may protect ovarian function during taxanes chemotherapy, while future explorations are needed to demonstrate the effectiveness. Although there are still many unknown and unclear problems to be solved about the taxanes’ reproductive toxicity, it is expected to provide some ideas for developing fertility preservation strategies in the future.

## Author contributions

MW and SXW conceived of the idea. CW performed the literature investigation and wrote the original draft of the manuscript. TW, DC, SMW, WT and LX designed the figures and table. JX, YH, YG and YC contributed to figure design and visualization. MW and SXW revised the manuscript. All authors contributed to the article and approved the submitted version.

## Funding

This work was financially supported by the National Natural Science Foundation of China (no. 81873824, 82001514) and the Fundamental Research Funds for the Central Universities (HSUT: 2021yjsCXCY087).

## Acknowledgments

Figures created with BioRender.com.

## Conflict of interest

The authors declare that the research was conducted in the absence of any commercial or financial relationships that could be construed as a potential conflict of interest. All authors contributed to the article and approved the submitted version.

## Publisher’s note

All claims expressed in this article are solely those of the authors and do not necessarily represent those of their affiliated organizations, or those of the publisher, the editors and the reviewers. Any product that may be evaluated in this article, or claim that may be made by its manufacturer, is not guaranteed or endorsed by the publisher.
